# The Book World of Medicine and Science

**Published:** 1894-05-19

**Authors:** 


### The Book World of Medicine and Science.

A Text Book of the Theory and Practice of Medicine. By American Teachers. Edited by William Pepper, M.D., LL.D. Vol. II. (Philadelphia: W. B. Saunders, 1894. pp. 1046).

If this work is to be taken as representing the standard of the medical teaching in Philadelphia we can most honestly congratulate that city on the educational facilities offered to its students. Throughout the book there are evidences of much editorial care, as well as of very complete grasp of their subjects by the various contributors, and almost everywhere the matter is brought thoroughly up to date. The writers are sufficiently numerous to enable each to speak with authority on his own subjuect, and yet not so large in number as to break the continuity of the work, and, as a result, we have a volume which is not only accurate in its descriptions, and in most cases reliable as a guide to treatment, but is thoroughly interesting from cover to cover. There are in it but few traces of mere conpilation and quotation: it is the authors themselves who speak to the reader, and, although the system of co-operative authorship adopted may occasionally lead to omissions and repetitions which would not occur in more systematic treatises, we doubt whether much has been overlooked which is of real importance.

As the result of a somewhat curious arrangement of its contents, Volume II. commences with a chapter on bacteria, infection, and immunity; seventy pages of very good description of a very large and important subject, but one belonging rather to the former volume and having but little relation to the matters treated of in that before us.

The chapter on rickets is good, and the advice that the food of a child during the first year of his life should, as far as possible, consist of mother's milk, is quite in accordance with general experience. The alternative offered when mother's milk cannot be obtained is not, however, as acceptable, and we may in all seriousness question the advice given as regards diet under such circumstances. Unless American milk is a very much stronger article than any we come across here, it can hardly bear mixture with four times its bulk of any diluent, nor can we believe that English experience would sanction the use of such a diluent as oatmeal gruel during the first weeks of life. As for the pharmaceutical treatment, which "consists largely in the administration of phosphorus," probably it is best avoided. The chapter on diabetes is not altogether satisfying. Most of the facts known regarding the disease are stated, but they are hardly made to stand in due perspective. For all he finds in it an ordinary reader might, for example, easily fail to estimate at its true value the difference between those cases which we all agree to call diabetes, and those which, although they so often end like true diabetes, are during their course so unlike the ordinary disease, as to have earned for themselves the non-committal appellation of glycosuria; nor does it appear that the author fully appreciates the very different value of dietetic treatment in these different forms, or the importance of not being too dietetic in some of the non-progressive cases common among people of arthritic tendencies, in whom the circulation of a little sugar is a far less evil than the attempt to remove the last trace of it by means of rigid diet.

No attempt is made to define gout, except so far as a definition may be deduced from its symptomatology. It is, however, spoken of as "the most conspicuous manifestation of the arthritic diathesis," which is described as "a peculiar and ill-defined retardation of nutrition," on which depend a large group of diseases, existing either in the same individual or among members of the same family who have derived their morbid predisposition from a common ancestral source. Besides gout he places in this category "eczema, impetigo, erythema, urticaria, and the allied mucous inflammations of the respiratory passages, together with chronic acid dyspepsia, congestion of the liver, lithiasis, hæmorrhoids, neuralgia hemicrania, congestive headache, acute and chronic muscular rheumatism, asthma, obesity, diabetes, and polyuria." This is probably as far as we can go with certainty. Lithæmia is perhaps rather synonymous with, than a cause of, the arthritic diathesis.

As regards the treatment of acute gout it is advised that at the outset the bowels should be cleared out by calomel and colocynth, or by a couple of grains of calomel at night, followed by one or two Seidlitz powders in the morning, and that when the local symptoms are fully displayed colchicum should be commenced, which, however, should not be carried to the extent of producing irritation of the stomach or purging.

While strongly advocating the bold use of salicylates in acute rheumatism, it is admitted that in certain cases they fail to give relief. Some of these cases may perhaps be of gonorrheal, or septic, or gouty origin; others merely febrile exacerbations of rheumatoid arthritis; but there is a residuum of truly rheumatic cases which are untouched by salicylates, and these, we are told, are best treated by quinine and alkaline salts, or in anæmic and adynamic patients by capsicum, guaiac, strychnine, arsenic, iron, and alcohol.

The chapter on diseases of the blood, contributed by Dr. Osler, is very good and complete. Among other things he insists on the desirability of carrying on the treatment of chlorosis until the hæmoglobin value, as shown by the hæmometric scale, is above 90 per cent. Under the administration of iron the distressing symptoms disappear long before the disease is overcome, and unless great perseverance be exercised, and the patient be kept under treatment for a much longer period than she is apt to think necessary, recurrence of symptoms is very likely to take place. We may perhaps dissent from the dogma that in chlorosis "the physician is a therapeutic master," for many cases of chlorosis obstinately refuse to recover under the administration of iron. Probably Dr. Osler would class these among cases of "toxic anæmia," but he does not say so, and curiously enough, although he refers to the suggestion that some forms of chlorosis are due to fæcal poisoning from constipation, he omits all reference to the use of aloes in their treatment.

The sections on diseases of the heart, contributed by Dr. Pepper, form a valuable treatise on that subject.

Many interesting details are given regarding the treatment of valvular disease and the accompanying muscular derangements, and among others reference is made to the utility of venæsection in cases where the venous system is so overfilled with blood and the cavities of the right heart so distended, that no amount of stimulation will restore the cardiac action until the intracardiac pressure is relieved. A couple of tracings are given showing the good effect of this proceeding when the dilated heart has lost its grip of the blood stream and fails to contract upon it.

In the chapter on diseases of the pleura we note that irrigation, after tapping for empyema, is, under any ordinary circumstances, both dangerous and unnecessary; only in putrid or gangrenous empyema is irrigation justifiable. For opening an empyema it is advised that a trocar and canula be used, sufficiently large to admit a soft catheter to act as a drain. The removal of a portion of the ribs is only advised when their retraction nips the tube, or when the lung fails to expand. It is stated that "cases due to simple infection by the pneumo-coccus, as shown by bacteriological examination of the pus, especially in infants, occasionally end in recovery after a single aspiration." The possibility of an empyema being caused by the pneumo-coccus, which is not specially a pus producing organism, should then be borne in mind.

Space prevents us from entering further into the contents of this very careful and useful book, which will doubtless become a work of daily reference to many practitioners.

